# On the representation of hierarchical structure: Revisiting Darwin’s musical protolanguage

**DOI:** 10.3389/fnhum.2022.1018708

**Published:** 2022-11-11

**Authors:** Shigeru Miyagawa, Analía Arévalo, Vitor A. Nóbrega

**Affiliations:** ^1^Department of Linguistics and Philosophy, Massachusetts Institute of Technology, Cambridge, MA, United States; ^2^Institute of Biosciences, University of São Paulo, São Paulo, Brazil; ^3^School of Medicine, University of São Paulo, São Paulo, Brazil; ^4^Institute of Romance Studies, University of Hamburg, Hamburg, Germany

**Keywords:** language, syntax, protolanguage, brain and language, birdsong, prosody

## Abstract

In this article, we address the tenability of Darwin’s musical protolanguage, arguing that a more compelling evolutionary scenario is one where a prosodic protolanguage is taken to be the preliminary step to represent the hierarchy involved in linguistic structures within a linear auditory signal. We hypothesize that the establishment of a prosodic protolanguage results from an enhancement of a rhythmic system that transformed linear signals into speech prosody, which in turn can mark syntactic hierarchical relations. To develop this claim, we explore the role of prosodic cues on the parsing of syntactic structures, as well as neuroscientific evidence connecting the evolutionary development of music and linguistic capacities. Finally, we entertain the assumption that the capacity to generate hierarchical structure might have developed as part of tool-making in human prehistory, and hence was established prior to the enhancement of a prosodic protolinguistic system.

## Introduction: Birdsong and language

Charles Darwin (1871, p. 55) noted that birdsong is the “nearest analogy to language.” Just as songbirds have an instinct to sing, humans have an instinct to speak, and both species display a pre-mastery stage: subsongs in birds and babbling in humans (Aronov et al., 2008). These correlations led Darwin to conjecture that, prior to language, our ancestors were singing to communicate, what Fitch calls “musical protolanguage” (Fitch, 2005, 2006, 2010, 2013).

Recent studies show a surprising parallel between language and birdsong beyond simply sharing a pre-mastery stage (Yip, 2006, 2013; Bolhuis et al., 2010; Bolhuis and Everaert, 2013; Moorman and Bolhuis, 2013; Samuels, 2015; Miyagawa, 2017). In observing juvenile zebra finches (*Taeniopygia guttata*), Liu et al. (2004) identified two learning strategies. In “serial repetition,” one syllable of the model is repeated and clearly articulated; in the motif strategy, the juvenile bird tries to imitate the tutor’s vocal display in its entirety, and the articulation is noisy and imprecise. Similarly, O’Grady (2005) and others note that a human infant may adopt either the “analytic” style, which produces clearly articulated, one-word utterances, or the “*gestalt*” style, which produces large chunks of speech that are poorly articulated.

Regions in the forebrain controlling vocal production have been identified in humans as well as three independent lineages of songbirds (e.g., zebra finches; Pfenning et al., 2014). These regions display convergent specializations in the expression of 50–70 genes per brain region. Furthermore, in birds that do not sing (e.g., chickens, *Gallus gallus domesticus*) and a primate that does not have language (e.g., macaques; *Macaca fuscata*), no direct projection connects the vocal motor cortex to brainstem vocal motor neurons (Belyk and Brown, 2017; Nevue et al., 2020). Such observations endorse the assumption that language and birdsong share a common neurobiological substrate (Cahill et al., 2021) that would have allowed auditory-vocal learning, a capacity necessary for linguistic competence to emerge (Jarvis, 2019).

Taking Darwin’s musical protolanguage as a starting point, we discuss the possible evolutionary scenario from a linear musical/rhythmic protolanguage to speech prosody that would develop into a full-fledged syntactic hierarchical system underlying language (de Rooij, 1975, 1976; Price et al., 1991; Schafer et al., 2000; Richards, 2010, 2016, 2017; Speer et al., 2011; Langus et al., 2012; a.o.). To develop this claim, we explore the role of prosodic cues on the parsing of syntactic structures, as well as neuroscientific evidence connecting the evolutionary development of musical and linguistic capacities. Finally, we entertain the assumption that the capacity to generate hierarchical structure might have developed as part of tool-making prior to language.

## Musical protolanguage

Like birdsong, Darwin (1871) assumed that the earliest musical protolanguage did not contain any propositional meaning. Birds sing to convey intention, typically the desire to mate (Marler, 1998, 2000; Berwick et al., 2011; Berwick et al., 2013; Bowling and Fitch, 2015). Darwin (1871, p. 56–57) conjectured that the musical protolanguage was for “charming the opposite sex.” Given the lack of meaning, this musical protolanguage by itself could not have developed into human language. Darwin suggested that our ancestors began to interweave gestures and sound imitations of other animals as precursors to words in order to insert meaning into the musical sequences.

In the same vein, but with more knowledge about human language than what was available to Darwin, Fitch (2005, 2010, 2013) suggests that for the musical protolanguage to have transformed into language, a second stage must have added “a fully propositional and intentional semantics” (2005:220; see also Fitch, 2004). Fitch suggested there was an integration of existing systems: the musical protolanguage and the propositional system. More specifically, Fitch’s version of a musical protolanguage expands Darwin’s original formulation by offering an account of how an intentional semantics —as opposed to lexical semantics— was assigned to melodic strings, as well as how modern humans developed advanced vocal control and learning; a major obstacle for a cohesive explanation on the phylogenetic history of a linguistic capacity. In this article, we argue instead that complex vocal control, which paved the way for singing and rhythmic utterances, might have enhanced a parsing mechanism for syntactic constituency, hence for the identification of hierarchic structures, by means of prosodic cues (e.g., pauses, prominence, nuclear stress, etc.). Fitch (2010, p. 499) also refers to his model as a “prosodic” protolanguage, which “[…] consisted of sung syllables, but *not* of notes that could be arranged in a scale, nor produced with a steady rhythm” (see also Fitch, 2006). His prosodic protolanguage model, however, focuses on the evolutionary development of prosodic units rather than on the impact of prosodic cues in the identification of syntactic hierarchical structure, as we are proposing.

**FIGURE 1:**
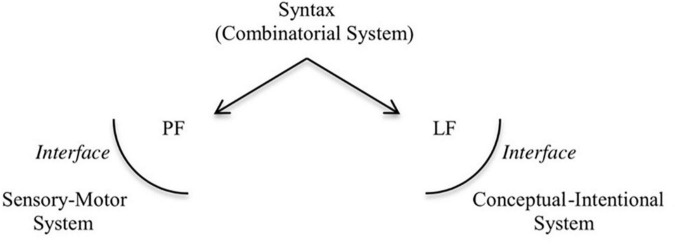
The architecture of the human language faculty.

Miyagawa et al. (2013, 2014) and Miyagawa (2017) note that components of human language existed long before language emerged.^1^ These components became integrated in recent evolutionary time, perhaps around 300–200 thousand years ago (kya) (Tattersall, 2008, 2010, 2012, 2016; Huybregts, 2017), to give form to language as we know it today. This integration of the musical protolanguage with the propositional component, as envisaged by Fitch, would have been a very complex process. Human language is associated with the core syntactic component, which generates structured phrases, and the interfaces to which the structured phrases are sent: the phonological form (PF), which connects to the sensory-motor system, and is responsible for the externalization of the structured phrases; and the logical form (LF), which connects to the conceptual-intentional system, assigning an interpretation to the structure (Chomsky, 1995, 2000; see Figure 1).

We argue that a prosodic protolanguage, resulting from complex vocal control ---fundamental for singing and rhythmic vocal displays---, would have been part of the PF component, enabling externalization of the core syntactic component. For this to happen, it developed the capacity to represent hierarchy within a linear signal. This proposal, when compared to Fitch’s, has the benefit of being more easily tested, since we can assess whether the absence of prosodic cues lead to divergent/unexpected parsing strategies or makes syntactic interpretation difficult.^2^ By pulling together research from neuroscience, primatology, and linguistics, we develop in this article a reasonably coherent picture of how hierarchy might have emerged in speech.^3^

One region that has been implicated in the creation of hierarchical relations is Broca’s area, specifically, the pars opercularis, or Brodmann area 44 (BA44) (Friederici et al., 2006; Friederici, 2009; Friederici et al., 2012; Kemmerer, 2012, 2015, 2021; Zaccarella and Friederici, 2015a,b,c). Studies have also explored the evolution of this region in humans and its homologs in other species, such as the great apes. These studies suggest that human BA44 is proportionately much larger than its homolog in other species (compared with the entire brain or specific regions like the entire frontal cortex; see Schenker et al., 2010; Smaers et al., 2017; Donahue et al., 2018), and that left BA44 in humans may have greater neuropil volume, suggesting greater space for local and inter-regional connectivity (Palomero-Gallagher and Zilles, 2019; Changeaux et al., 2021). We explore the idea that if the musical protolanguage played a role in the evolution of language by transforming into what we call speech prosody, as Darwin originally suggested, it may have involved BA44 and its critical connections to other regions.^4^

## Prosody

Words in language are uttered in a linear fashion. The words are not simply linearly ordered but are also hierarchically organized, and this hierarchy comprises the essential component for associating meaning to the expression. The hierarchy itself is an abstract representation, and is commonly communicated by prosody, as a layer of supra-segmental phonological information on top of the string of words (e.g., Selkirk, 1986; Jackendoff, 1997; Büring, 2013). There are two types of prosody: emotional and linguistic. Emotional prosody signals the speaker’s emotional state or the emotional content of the expression, while linguistic prosody signals syntactic structure and thematic relations.^5^ Here we will focus on the latter. We give three examples of such prosody: (i) pauses, which mark clausal structure, (ii) relative prominence assigned to units within a noun phrase, and (iii) nuclear stress, which is assigned within a verb phrase.

### Pause

The following shows how pause, or major prosodic constituents, can be placed within a sentence (from Büring, 2013, p. 865).

**Table:** 

(1)	when Roger left the house became irrelevant.
	(a) when Roger left [PAUSE] the house became irrelevant
	(b) when Roger left the house [PAUSE] became irrelevant

(1) shows how pauses indicate structural boundaries. The silent intervals in (1a) and (1b) signal the end of a subordinate clause, with the varying positions leading to different interpretations.^6^

### Prominence: Noun phrase

Speakers can tell which syllable is prominent in an utterance. Prominence can often be measured by duration, intensity, fundamental frequency (pitch) and other acoustic measures. Prominent syllables tend to be longer and louder. So, a syllable (along with the word that contains it) is perceived as prominent if it is in the location of the local maximum in the fundamental frequency curve. Conversely, it is perceived as less prominent if it is in the location of the local minimum in the fundamental frequency curve (see Büring, 2013, and references therein). In English, very roughly, the last syllable/word in a constituent receives relative prominence (e.g., Selkirk, 1986). The following is modeled on similar examples from Büring (2013).

**Table:** 

(2)	a.				(*)
			(*)		(*)
		(*)	(*)		(*)
		fancy	shirt	and	slacks

**Table:** 

					(*)
		(*)	(*)		(*)
b.		tie,	shirt	and	slacks

The number of asterisks indicates relative prominence. In (2a), *fancy* and *shirt* differ in prominence, with *shirt* receiving more prominence. This indicates that *shirt* is at the right edge of the phrase that also contains *fancy*. The third word, *slacks*, receives more prominence than *shirt*, indicating that it is at the right edge of another phrase.

(3) [[fancy shirt] and slacks]

This is a hierarchical relation, with *fancy shirt* in the lower tier of the hierarchy.

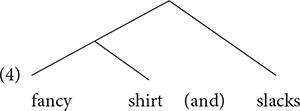

In (2b), no distinction exists between *tie* and *shirt*, so these words do not constitute a phrase. The relative prominence of the last word, *slacks*, shows that this word is on the right edge of the entire phrase: [*tie shirt and slacks*].

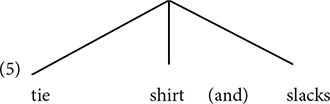

### Prominence: Nuclear stress rule

Within a verb phrase of a sentence with neutral focus, a rhythmically prominent stress falls on a particular constituent, called Nuclear Stress (NS) (Chomsky and Halle, 1968; see also Zubizarreta, 1998; Reinhart, 2006). The NS in the example below falls on *book*, the final element in the verb phrase (and the sentence).

(6) Mary read a book.

There is general recognition that syntactic structure plays a crucial role in the assignment of NS (e.g., Chomsky, 1971; Jackendoff, 1972; Cinque, 1993; Selkirk, 1995; Kahnemuyipour, 2004, 2009; Reinhart, 2006; Truckenbrodt, 2006; Kratzer and Selkirk, 2007; Féry, 2011). It appears at first that the NS is assigned to the last element in the sentence. This would be a linearly based analysis of NS. A key observation for the structurally based NS assignment is that in a language such as German, where the object precedes the verb, the NS falls not on the final element, but on the object, just as in English.

**Table:** 

(7)	*Hans hat ein* ***Buch*** *gelesen*.
	Hans has a book read
	“Hans has read a book.”

In either order, English or German, the verb and the object are in the verb phrase: [_VP_ Verb OBJ]. There is an assumption that the verb must vacate the verb phrase and move to a higher position, leaving, in this case, only the object: [_VP_ __ OBJ]. Is it always the object that is assigned the NS? The example below shows that it is not.

(8) Mary read a book about the **moon**.

The NS in (8) falls on *moon* within the prepositional phrase that follows the object. This indicates that the NS is assigned to the highest element in the verb phrase (Kahnemuyipour, 2004, 2009; Kratzer and Selkirk, 2007).

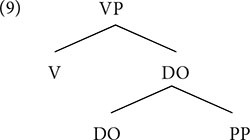

  The NS assignment is not dependent on linear order, but strictly on hierarchical structure. In this way, speech prosody marks hierarchy.^7^

## Music and prosody

Some evolutionary theories contend that music and language have a common progenitor that gave rise to an early communication system (Brown, 2001; Mithen, 2005). Both human speech and music contain prosody, which in turn contains melody (intonation) and rhythm (stress and timing) (Nooteboom, 1997; see also Yip, 2013). Music and prosody have been shown to recruit overlapping neural regions, supporting Darwin’s original idea and the evolutionary theories that it spawned (Peretz et al., 1994; Patel, 2008, 2012). Some have suggested that language and music are on a continuum, without a sharp line of demarcation (Jackendoff, 2009; Patel, 2010; Koelsch, 2012). Early in life, infant-directed speech (IDS), or “motherese” (Gleitman et al., 1984; Bates et al., 1995; de Boysson-Bardies, 1999) seems to imitate song, and infants show overlapping neural activity to IDS and instrumental music (Kotilahti et al., 2010).

In studies of amusia without aphasia, Patel et al. (1998) observed that prosodic and musical discrimination were preserved or affected together, suggesting that the perception of prosody and musical contour share overlapping cognitive and neural resources.^8^ Furthermore, studies showing that individuals with a congenital deficit in music perception typically also exhibit deficits in perception of pitch in language (Peretz, 1993; Liu et al., 2010; Nan et al., 2010; Tillmann et al., 2011).

Over the last several decades, melodic intonation therapy (MIT) has been used to improve language production in patients with aphasia. Often, these patients have global aphasia and respond poorly to other forms of classical therapies. Patients who benefit from MIT may be activating remaining frontoparietal networks critical to language, music and motor processing (Sparks et al., 1974; Leonardi et al., 2017).

According to Hausen et al. (2013), studies using fMRI have shown that music and language recruit overlapping neural regions, including superior, anterior and posterior temporal, parietal, and inferior frontal areas (Koelsch et al., 2002; Tillmann et al., 2003; Brown and Martinez, 2007; Rauschecker and Scott, 2009; Schön et al., 2010; Abrams et al., 2011; Rogalsky et al., 2011).

While music and prosody are largely processed in the right hemisphere of the brain (Weintraub et al., 1981; Bradvik et al., 1991), hierarchy is associated with left Broca’s area (BA44) (Friederici et al., 2006; Friederici, 2009; Friederici et al., 2012; Zaccarella and Friederici, 2015a,b,c). Meyer et al. (2002) showed that speech normally recruits both hemispheres, while prosodic speech without any segmental information activates mostly the right hemisphere. Speech processing streams connect the hemispheres via the posterior portion of the corpus callosum. As evidence of this, syntax-prosody mismatches in an ERP paradigm did not elicit an anterior negativity in patients with lesions to the posterior third of the corpus callosum (vs. patients with lesions to the anterior two-thirds of the corpus callosum and controls) (Sammler et al., 2010).

## Stone tools: Source of hierarchy?

If BA44 is a critical piece of the puzzle when it comes to generating hierarchy, then presumably the original musical protolanguage would have undergone enhancement by connecting to this region to produce speech prosody. Under this view, the capacity to generate hierarchical structures existed prior to the enhancement. If so, how did the capacity to generate hierarchical structure develop? One view is that hierarchical cognition developed as part of tool-making, as initially suggested by Lashley (1951), and recently expanded by Fitch and Martins (2014), Asano and Boeckx (2015), and Asano (2021). This idea, which is controversial (Putt et al., 2017), was primarily developed by Greenfield’s grammars of action (Greenfield, 1991, 1998). From their studies with non-human primates, Greenfield and colleagues suggested three general “grammatical” strategies: pairing strategy, pot strategy, and subassembly strategy; this last one, subassembly, requires hierarchical organization of information. They observed that while non-human primates could engage in the first two strategies, only humans are capable of the third strategy, suggesting hierarchical organization is an exclusively human trait.

A large body of work has applied this general approach to stone tools, with the assumption that higher cognitive functions in modern humans are linked with the evolution of motor control (Lieberman, 2006; see also Holloway, 1969; Wynn, 1991; Fitch and Martins, 2014). Stone tools are made from flake units, which are combined to form assemblies, and these assemblies make up the tool’s higher-order architecture (Miller et al., 1960). Earlier (i.e., Pleistocene era) tools do not evidence this kind of hierarchical structure. Moore (2010) argues that it appeared in late Middle Pleistocene, around 270 kya, when the Mousterian style of tool-making appeared with the Neanderthals; however, rudimentary hierarchical cognition may have supported tool-making much earlier, approximately 800 kya or earlier, during the Acheulean phase (Moore, 2010; Stout and Hecht, 2014; Gaucherel and Noûs, 2020).^9^ If true, the capacity for hierarchical cognition existed long before human language emerged. If so, this baseline would have allowed the musical protolanguage to evolve and give rise to speech prosody. Additional support for these ideas comes from imaging studies showing overlapping activations for language and tool use tasks (Stout et al., 2008; Higuchi et al., 2009; Stout and Chaminade, 2012; Osiurak et al., 2021).^10^

## What came first?

In this article, we traced our arguments beginning with Darwin’s original suggestion that “[…] musical cries by articulate sounds may have given rise to words expressive of various complex emotions” (Darwin, 1871; see also Oesch, 2020). This statement implies the following sequence of emerging functions: isolated melodic cries, then complex vocalizations (with increasing articulatory refinement), then simple linguistic utterances, followed by increasingly complex language containing words capable of conveying emotions. A parallel theory suggests music and language may have evolved simultaneously on a spectrum (Morley, 2013; Oesch, 2019). This last theory gains strength in the fact that fossil records —the only direct source of information on this matter— are inherently limited, which currently precludes us from determining causality.

Thus, given these limitations, an equally plausible proposal would be the reverse: that speech in fact preceded music. Here we list a few arguments that make this possibility less convincing. As mentioned above, studies have revealed an expansion of several cortical regions (e.g., BA44, auditory-vocal cortical regions) as well as sensorimotor connectivity in humans relative to non-human primates, which is thought to have permitted the enhancement of critical components of language, including vocal working memory and vocal repertoire size (Schenker et al., 2010; Smaers et al., 2017; Aboitiz, 2018; Donahue et al., 2018; Ardesch et al., 2019; Palomero-Gallagher and Zilles, 2019; Changeaux et al., 2021). Compared with non-human primates and other species known to engage in ‘‘cooperative vocal turn-taking,’’^11^ humans arguably have the most complex language, at least in terms of vocabulary size and internal structure. Thus, the work in comparative neuroanatomy and connectivity would suggest that language, at least in its most evolved, modern state, would not have emerged earlier than musical abilities.

Although archeologists have suggested that the fine motor control required for modern-day vocalizations may have been present in *Homo heidelbergensis* as early as 5–800,000 years ago (MacLarnon and Hewitt, 1999; Martinez et al., 2013; Oesch, 2019), some forms of musical expressions, such as drumming or marking a beat (e.g., beat entrainment), do not require any vocalizations at all. So, in line with the above arguments, the evolutionary record would suggest that the biological substrates and mechanisms required for music production would have been in place before those for the most advanced forms of language. However, several authors have argued that beat entrainment requires fine motor control, including vocal control (see Patel, 2021; Shilton, 2022).^12^ With this in mind, we can speculate that until fine motor control and vocalization systems to support musical as well as linguistic communication emerged in early hominins, it is very likely that gestures might have played an even more prominent role in communication.

So, if the fossil record is limited, what can other lines of research contribute to elucidating these questions? One hope lies in modern neuroscientific research. As our technologies advance at unprecedented rates, well-designed studies using connectivity, electrophysiology, electrocorticography, and coherence should test musical and language processing in humans as well as other species. As we become progressively closer to understanding the real time processes involved in different forms of musical and linguistic processing, we can further our understanding of how evolutionarily more recent structures may have supported such processes, thus providing evidence for or against theories tracing the sequential or parallel emergence of these skills.

## Concluding remarks

Darwin’s musical protolanguage, if it existed, must have undergone many critical changes before it became modern-day language. One crucial step would have been tapping into the ability to produce hierarchical structure, which is only present in human language. We suggest that this step involved enhancement of the musical system to transform it to speech prosody, which can mark hierarchical relations. Other steps were needed for the hierarchical structure marked by prosody to link up with a fully propositional intentional semantics. But it is a crucial step, as we can see by the pervasive nature of hierarchical structure in human language.

## Data availability statement

The original contributions presented in this study are included in the article/supplementary material, further inquiries can be directed to the corresponding author.

## Author contributions

All authors listed have made a substantial, direct, and intellectual contribution to the work, and approved it for publication.

## Footnotes

1 See also Fitch (2002) and Hauser et al. (2002).

2 For example, Sandler et al. (2011) stress the role of prosodic cues in the development of syntactic complexity when analyzing the development of the Al-Sayyid Bedouin emergent sign language. In this language, rhythmic and facial cues are directly aligned at constituent boundaries. The importance of prosodic cues in the development of syntactic constituency can further be tested in other nascent linguistic system that lack any previous linguistic bias, such as the Cena rural sign language in Northeast Brazil (Almeida-Silva and Nevins, 2020).

3 It is relevant to point out that Benítez-Burraco and Elvira-García (2022) reach similar conclusions by exploring the role of self-domestication in the evolutionary development of speech prosody. In their view prosody, which is argued to have been affected by human self-domestication, might have favored syntactic complexification through a series of bootstrapping effects.

4 Katz and Pesetsky (2011) and Roberts (2012) show that both music and language employ a parallel computation for hierarchical structure building. We acknowledge that the cognitive mechanisms underlying hierarchical structure in both music and language might have had a common ancestry, as will be explored later (see also Jackendoff, 2009; Boeckx and Fujita, 2014; Fitch and Martins, 2014; Asano and Boeckx, 2015; Asano, 2021; Asano et al., 2022).

5 Prosody often marks structure in neutral focus. If there is narrow focus in way of stress for emphasis, prosody does not necessarily mirror the structure of the expression (Ladd, 2008). Some languages, however, seem to involve a different pattern. Shanghainese and some Bantu languages display a mismatch between prosody and syntactic structure in neutral focus (e.g., Zubizarreta, 2009; Han et al., 2013). This linguistic variability with respect to prosodically marked neutral focus led some linguists to suggest that prosody may not have a faithful one-to-one mapping from syntax, being responsible for mapping only certain syntactic domains (Selkirk, 2009, 2011).

6 Yip (2013, p. 191) indicates that a “motif” could be roughly equated to a phrase, “in its tendency to be surrounded by ‘pauses”’. Such category in birdsong plays a crucial role during ontogeny, since infants first begin copying small chunks of the target song. Williams and Staples (1992) show that the chunk boundaries produced by infants correlate with the silent interval delimited by the pauses circumscribing a motif, suggesting that similar acoustic cues assist on the identification of the internal structure of a song, facilitating its segmentation — a strategy that is parallel to the prosodic bootstraps in language acquisition (Yip, 2013; Mol et al., 2017). Song segmentation, however, seems to be circumscribed to the identification of which note strings might comprise a motif, and which are the linear organizations of motifs into a complete song. Birdsong involves a finite-state mechanism to combine notes into motifs, and motifs into songs (Berwick et al., 2011, Berwick et al., 2012). A finite-state mechanism resorts to strictly sequential steps (linear probability), hence lacks hierarchical organization. The latter is only available in combinatorial systems that demand a more powerful working memory, such as context-free or context-sensitive systems (Joshi, 1985), which was not observed in songbirds.

7 Further prosodic phenomena responsible for marking constituent boundaries are (i) stress prominence in English, which normally falls on the rightmost constituent within a phrase (e.g., [[*A sènator* [*from Chicágo*]] [*wòn* [*the làst eléction*]]] (Chomsky and Halle, 1968 *apud* <*ref*> Selkirk, 2011, p. 435), (ii) *liason* in French, i.e., maintenance of a word-final consonant before a vowel, [[Le peti**t** **â**ne] [le suivait]] “The little donkey followed him” vs. [[*Le peti*] [[*aime*] [*le Guignol*]] “The little one loves the puppet theater” (Selkirk, 1974 *apud* Selkirk, 2011, p. 435– 436). Several additional phenomena can be found in Selkirk (2011). In sign languages, non-manual markers, such as head position and facial expression, serve the role of prosodic cues, and are equally relevant for syntactic parsing involved in topicalization, relative clauses, and *wh*-constructions (see Baker and Padden, 1978; Liddell, 1978, 1980; Neidle et al., 2000, for American Sign Language).

8 Earlier studies have reported a dissociation between the processing of language and music (Marin, 1989; Peretz and Morais, 1989, 1993; Sergent, 1993). See Patel (2012) for comments on this apparent dissociation.

9 More specifically, Moore (2010) shows that hierarchical flaking is necessary for stone tool types that demand multiple preparatory steps prior to a flake removal, such as Acheulean bifaces and the Levallois method. The production of Oldowan choppers, differently from bifaces and the Levallois’ core preparation, only requires the extraction of high mass from the core, lacking preparatory flaking (see also Stout, 2011; Stout et al., 2018, for similar conclusions).

10 It is relevant to point out that vocal learning and vocal control evolved independently from language (Jarvis, 2004, Jarvis, 2019), hence prior to syntactic structuring. We also find suggestive evidence that hierarchy was presumably co-opted from the abilities involved in the motor actions of stone tool-making (see Fitch and Martins, 2014; Asano and Boeckx, 2015; Asano, 2021). With this timeline in mind, we can entertain an evolutionary scenario where complex vocal control, roughly understood as an embryonic stage of prosodic cues, might have enhanced the representation of hierarchic structure in the expressive utterances of early human, gradually leading to present-day syntax. In this scenario, we can say that prosody and syntactic structuring co-evolved.

11 According to Oesch (2019), these are a rare type of vocalization that bridges the gap between animal calls and human speech.

12 We thank one of the reviewers for suggesting us this point.
